# Targeting Ubiquitin–Proteasome System With Copper Complexes for Cancer Therapy

**DOI:** 10.3389/fmolb.2021.649151

**Published:** 2021-04-13

**Authors:** Xin Chen, Q. Ping Dou, Jinbao Liu, Daolin Tang

**Affiliations:** ^1^ Guangzhou Municipal and Guangdong Provincial Key Laboratory of Protein Modification and Degradation, Affiliated Cancer Hospital & Institute of Guangzhou Medical University, School of Basic Medical Sciences, Guangzhou Medical University, Guangzhou, China; ^2^ Department of Oncology, School of Medicine, Barbara Ann Karmanos Cancer Institute, Wayne State University, Detroit, MI, United States; ^3^ Departments of Pharmacology & Pathology, School of Medicine, Wayne State University, Detroit, MI, United States; ^4^ Department of Surgery, UT Southwestern Medical Center, Dallas, TX, United States

**Keywords:** copper complex, cancer, ubiquitin, proteasome, degradation

## Abstract

Characterizing mechanisms of protein homeostasis, a process of balancing between protein synthesis and protein degradation, is important for understanding the potential causes of human diseases. The ubiquitin–proteasome system (UPS) is a well-studied mechanism of protein catabolism, which is responsible for eliminating misfolded, damaged, or aging proteins, thereby maintaining quality and quantity of cellular proteins. The UPS is composed of multiple components, including a series of enzymes (E1, E2, E3, and deubiquitinase [DUB]) and 26S proteasome (19S regulatory particles + 20S core particle). An impaired UPS pathway is involved in multiple diseases, including cancer. Several proteasome inhibitors, such as bortezomib, carfilzomib, and ixazomib, are approved to treat patients with certain cancers. However, their applications are limited by side effects, drug resistance, and drug–drug interactions observed in their clinical processes. To overcome these shortcomings, alternative UPS inhibitors have been searched for in many fields. Copper complexes (e.g., CuET, CuHQ, CuCQ, CuPDTC, CuPT, and CuHK) are found to be able to inhibit a core component of the UPS machinery, such as 20S proteasome, 19S DUBs, and NPLOC4/NPL4 complex, and are proposed to be one class of metal-based anticancer drugs. In this review, we will summarize functions and applications of copper complexes in a concise perspective, with a focus on connections between the UPS and cancer.

## Introduction

**FIGURE 1:**
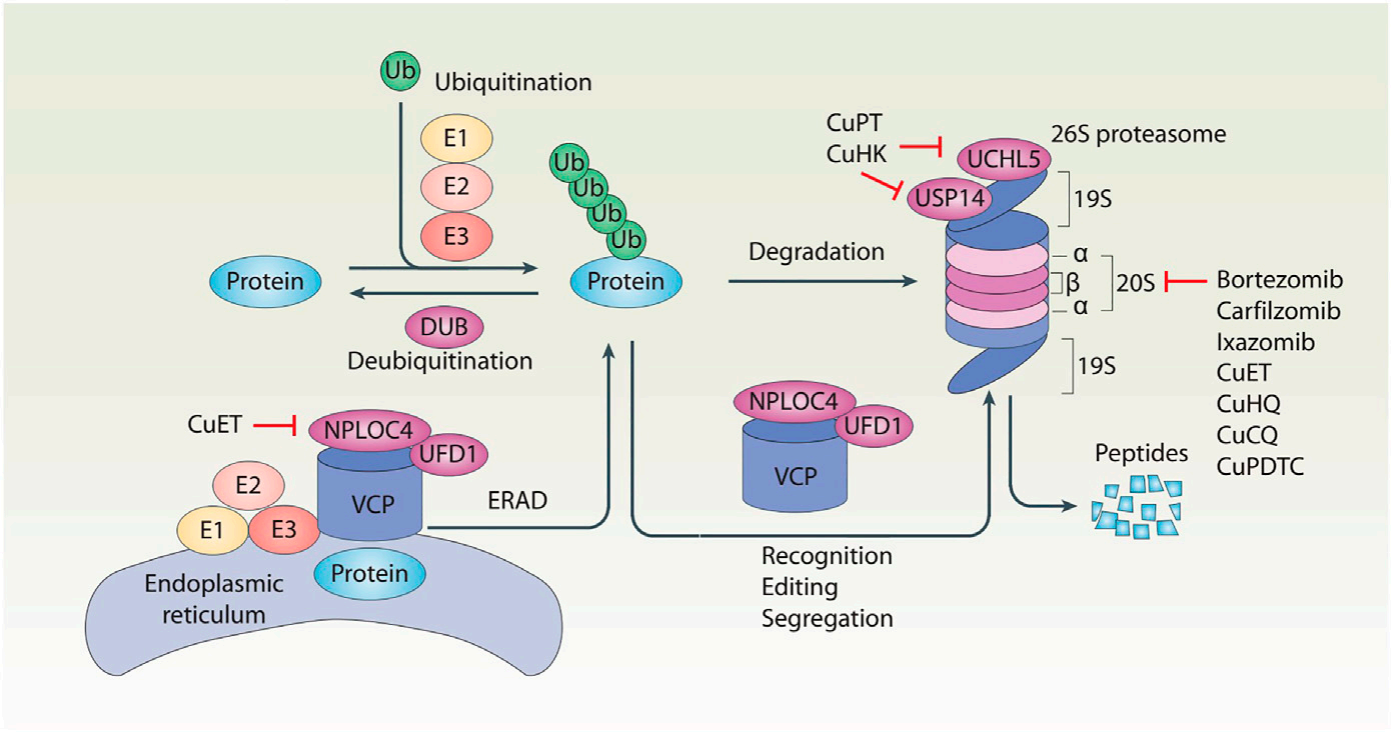
Schematic of the ubiquitin–proteasome system of protein degradation. In the ubiquitin–proteasome system, ubiquitination and deubiquitination are two reversible events that control protein levels. Generally, ubiquitination is the addition of ubiquitin (Ub) molecules to lysine residues of a protein by a cascade of enzymes (E1, E2, and E3), leading to the degradation of the substrate proteins in the 26S proteasome. In contrast, deubiquitination is catalyzed by cytosolic deubiquitinase (DUBs) or proteasomal DUBs (e.g., UCHL5 and USP14) to remove Ub from ubiquitinated proteins. In addition, the VCP–UFD1–NPLOC4 complex not only participates in the recognition, editing, and segregation of ubiquitinated substrates, but also plays a unique role in the regulation of endoplasmic reticulum-associated degradation (ERAD) by extracting substrates for degradation in the cytoplasmic proteasome.

Protein, a complex molecule composed of amino acids, is the basic component of all living organisms. Protein homeostasis is a dynamic balance between protein synthesis and protein degradation, which is critical for maintaining healthy cell functions. In contrast, altered protein homeostasis may produce misfolded, aggregated, and mutated proteins, which are implicated in various pathological conditions and diseases, including cancer (Bastola et al., 2018). Although half-lives of different proteins may vary greatly, the turnover rate of a protein is usually related to its function or subcellular location. The ubiquitin–proteasome system (UPS) and autophagy play major roles in degradations of short-lived and long-lived proteins, respectively (Bachmair and Varshavsky, 1989; Kuma et al., 2004). Autophagy relies on formation of different membrane structures to engulf and destroy cargos (including proteins and organelles), whereas the UPS is mainly carried out to degrade proteins through a hierarchical enzymatic cascade. Both autophagy and the UPS constitute potential targets for cancer therapy. In particular, proteasome inhibitors bortezomib, carfilzomib, and ixazomib were approved by the US Food and Drug Administration (FDA) for treatment of multiple myeloma in 2003, 2012, and 2015, respectively (Manasanch and Orlowski, 2017). However, side effects and secondary drug resistance of these proteasome inhibitors limit their wide applications (Brunnert et al., 2019; Ge et al., 2020). These challenges highlight an urgent need for development of novel UPS inhibitors for cancer patients. In this mini-review, we first describe key components of the UPS (Figure 1), and then discuss potential implication of copper complexes as new UPS inhibitors in cancer treatment.

## Ubiquitin–Proteasome System: A Complexed but Coordinated System

The UPS is a highly controlled mechanism of protein degradation and turnover in cells, starting with monomeric ubiquitin, a small protein with a molecular mass of approximately 8 kDa (Ciehanover et al., 1978; Johnson et al., 1995). Ubiquitin is first activated by E1 in an adenosine triphosphate (ATP)–dependent manner. Activated ubiquitin is then conjugated by an E2 enzyme, resulting in transfer of ubiquitin to an internal lysine of the target protein via one of E3 ligases. Subsequently, through an ATP-dependent process, the polyubiquitin–protein conjugate is degraded by the 26S proteasome complex composed of two subcomplexes, the 20S proteasome and the 19S regulatory particle. Disassembly of the 26S complexes into the 20S proteasomes and the 19S regulatory particle can be caused by diminished ATP (Hoglinger et al., 2003), aspartate deficiency (Meul et al., 2020), or elevated reactive oxygen species (ROS) levels (Livnat-Levanon et al., 2014). Finally, an abnormal protein is cleaved into short peptide fragments, and the polyubiquitin chain is released and is trimmed into monomeric ubiquitin by deubiquitinating enzymes (DUBs) (Mevissen and Komander, 2017). Of note, the E3 ubiquitin ligase is responsible for substrate recognition and more than 600 putative E3 ligases determine the diversity of the UPS.

## The Proteasome as a Target for Cancer Therapy

The 20S proteasome is a 700-kDa large complex formed by four stacked rings, two outer α-rings and two inner β-rings. Each α-ring or β-ring is formed by seven subunits. The constitutive active sites are β1, β2, and β5 that are responsible for caspase-like (C-like), trypsin-like (T-like), and chymotryptic activity (CT-like), respectively. The 19S regulatory particle plays essential roles in processing ubiquitylated substrates by binding, deubiquitinating, and unfolding ubiquitinated proteins (Liu and Jacobson, 2013). Substrate recognition is mediated by ubiquitin receptors (e.g., proteasome 26S subunits non-ATPase 2 [PSMD2/RPN1] and non-ATPase 4 [PSMD4/RPN10]and adhesion regulating molecule 1 [ADRM1/RPN13]) located in the 19S regulatory particle (Martinez-Fonts et al., 2020). In order to pass through a narrow channel in the 20S proteasome, domains within target proteins must be unfolded before entering. ATPases of the 19S regulatory particle exhibit conformational changes and function in gate opening, which is required for the degradation of a specific protein (Smith et al., 2011).

The proteasome has been demonstrated as a potential therapeutic target for cancer. Specially, 20S proteasome inhibitors bortezomib, carfilzomib, and ixazomib, which mainly target the CT-like catalytic subunit of the proteasome β5 subunit, are approved to treat patients with certain cancers, such as multiple myeloma. Although the precision mechanism remains obscure, several pathways are involved in proteasome inhibitor-induced tumor suppression. Proteasome inhibition results in stabilization of several key tumor suppressing proteins (e.g., NFKB inhibitor alpha [NFKBIA/IKBA] (Chen et al., 1995), bcl2-associated X, apoptosis regulator [BAX] (Li and Dou, 2000), cyclin-dependent kinase inhibitor 1A [CDKN1A/p21] (Bloom et al., 2003), cyclin-dependent kinase inhibitor 1B [CDKN1B/p27] (Pagano et al., 1995), and tumor protein p53 [TP53] (Lopes et al., 1997)), which are responsible for cell growth inhibition or cell death induction. In addition, inhibition of the proteasome causes the accumulation of unfolded and misfolded proteins, which triggers unfolded protein response (UPR), endoplasmic reticulum (ER) stress, and apoptosis (Obeng et al., 2006). However, severe side effects and drug resistance observed in clinical process of these applications demand discovery of more specific, less toxic proteasome inhibitors.

## Targeting DUBs: A Novel Strategy for Cancer Therapy

DUBs can be divided into six families based on their different structures: ubiquitin C-terminal hydrolases (UCHs), ubiquitin-specific proteases (USPs), ovarian-tumor proteases (OTUs), JAMM/MPN domain–associated metallopeptidases (JAMMs), Machado–Joseph disease protein domain proteases (MJD), and monocyte chemotactic protein-induced protein (MCPIP) (Mevissen and Komander, 2017). Three DUBs are associated with the proteasome, two cysteine proteases [ubiquitin C-terminal hydrolase L5 (UCHL5/UCH37) and ubiquitin-specific peptidase 14 (USP14)], and a proteasome 26S subunit [non-ATPase 14 (PSMD14/RPN11)]. The Zn(II) metallo-protease PSMD14 is an integral subunit of the 19S regulatory particles, whereas the activities of UCHL5 and USP14 can be enhanced when they were recruited to the 19S regulatory particles (D'Arcy et al., 2015). USP14 binds to PSMD2, while UCHL5 can form a complex with PSMD4 and ADRM1 (Leggett et al., 2002; Stone et al., 2004; Qiu et al., 2006). Proteasomal DUBs may play different and overlapping functions in inhibiting ubiquitination. For example, UCHL5 or USP14 removes the polyubiquitin chain from the distal end of target protein, whereas PSMD14 cuts the entire ubiquitin chain at the base of the polyubiquitin chain during degradation process (Lam et al., 1997; Verma et al., 2002; Yao and Cohen, 2002). Binding of ubiquitinated proteins to USP14 causes 20S proteasome gate opening, thereby linking proteasomal deubiquitination to protein degradation (Peth et al., 2009). Indeed, knockdown of UCHL5 or USP14 alone increases the rate of proteasomal protein degradation but fails to affect structure and proteolytic capacity of the proteasome (Hu et al., 2005; Yao et al., 2006). However, these findings are challenged by a recent study that the loss of UCHL5 activity also impairs global protein turnover (Deol et al., 2020).

Accumulating evidence suggests a protumor role of proteasomal DUBs in different cancers. For example, UCHL5 accelerates growth of endometrial cancer by activating the WNT-catenin beta 1 (CTNNB1) pathway (Liu et al., 2020). UCHL5 positively regulates Hedgehog signaling by removing ubiquitination of Smoothened, Frizzled class receptor (SMO) protein in cancer cells (Zhou et al., 2018). *In vitro* and *in vivo* studies have shown that genetic and pharmacological inhibition of USP14 induces degradation of an androgen receptor (AR), favoring tumor suppression in AR-positive breast cancer cells or prostate cancer cells (Liao et al., 2017; Liao et al., 2018). USP14 promotes tumor growth by regulating DNA damage response or ER stress-mediated autophagy in non-small cell lung cancer cells (Moghadami et al., 2020; Sharma and Almasan, 2020). Alone this line, several compounds targeting UCHL5 and USP14 have been developed, including b-AP15 (D'Arcy et al., 2011), VLX1570 (b-AP15 analog) (Wang et al., 2016), AC17 (curcumin analog) (Zhou et al., 2013), and various metal-based complexes (Liu et al., 2014b; Chen et al., 2018a; Li et al., 2019a). Inhibition of deubiquitinating activity of USP14 and UCHL5 by b-AP15 rescues the protein level of TP53 in tumors from *Tp53* ^−/−^ mice (Ma et al., 2020). VLX1570 or b-AP15 inhibits tumor migration and induces apoptosis in diffuse large B-cell lymphoma, prostate cancer, and Ewing sarcoma cells (Shukla et al., 2016; Cai et al., 2017; Jiang et al., 2019). Importantly, a phase I study showed that VLX1570 has antitumor effects in patients with multiple myeloma, although two patients developed severe pulmonary toxicity (Rowinsky et al., 2020). Similar to proteasome inhibitors, efforts are needed to develop specific DUB inhibitors with greater therapeutic effects and less toxic side effects.

## ER-Associated Degradation: A Quality-Controlled Degradation of Misfolded Proteins

Disruption of protein folding in ER activates UPR or ER-associated degradation (ERAD) to eliminate misfolded proteins. Unlike UPR, ERAD is a quality control process of ubiquitination of misfolded proteins in ER and subsequent proteasome degradation. Although signal and modulation of ERAD is still poorly understood, it has been shown that the valosin-containing protein (VCP/CDC48/p97)–ubiquitin recognition factor in ER-associated degradation 1 (UFD1)-NPL4 homolog, ubiquitin recognition factor (NPLOC4) complex, may play a major role in blocking activation of ERAD. VCP is a conserved chaperone-like ATPase associated with protein unfolding activities by translocating ubiquitinated substrates through its central pore, which is formed by a homohexameric, ring-shaped complex. In addition to ERAD, VCP also participates in regulation of the mitochondria-associated degradation pathway and quality control on ribosomes translating cytosolic proteins (Forster et al., 2014). VCP commonly collaborates with heterodimeric cofactors UFD1 and NPLOC4. Hyperosmotic stress induces phase separation of proteasome-containing nuclear foci (containing ubiquitinated proteins, VCP, and several proteasome-related proteins), which collectively constitute a proteolytic site (Yasuda et al., 2020). Compared to normal cells, cancer cells are characterized with upregulation of the VCP-UFD1-NPLOC4 pathway to digest false-synthesized and misfolded proteins (Tsujimoto et al., 2004; Yamamoto et al., 2004; Huiting et al., 2018; Lu et al., 2019). Thus, inhibition of the VCP-UFD1-NPLOC4 pathway may represent as a selective treatment option for cancer.

## Copper Complexes as UPS Inhibitors in Cancer Treatment

Copper is an essential transition metal ion and plays an important role in maintaining normal cellular function (Linder and Hazegh-Azam, 1996). Excessive copper ions (Cu(II)) may generate ROS, which mediates oxidative damage to lipids, proteins, and DNA. Multiple types of tumors (e.g., breast and colorectal cancers) have aberrantly elevated copper levels, which promote tumor progression by increasing cell proliferation and stimulating angiogenesis and metastasis (Denoyer et al., 2015; Li, 2020; Shanbhag et al., 2020). This abnormal copper pathway in cancer can be targeted by two strategies. On one hand, a copper chelator can be used to inhibit the pro-survival effect of copper in cancer cells (Gupte and Mumper, 2009). On the other hand, using copper ionophores or copper complexes to increase intracellular copper levels could also suppress tumor growth (Wehbe et al., 2017).

In addition to mediating oxidative damage, several lines of evidence emphasize selective role of copper complexes as potential UPS inhibitors, which contribute to their anticancer activities. First, addition of Cu(II), but not other metal ions [e.g., Zn(II), Ni(II), and Al(III)] and Cd(II), to protein samples decreases the thermal stability of ubiquitin (Milardi et al., 2007). Second, incubation with Cu(II) leads to formation of spherical ubiquitin aggregation, and this process is inhibited by Cu(II) chelation or reduction to Cu(I) (Arnesano et al., 2009). Third, Cu(II) at micromolar concentration inhibits all three kinds of proteasomes activities (Santoro et al., 2016). Fourth, in a cell-free system, Cu(II) impairs channel gating of the 20S proteasome, but does not catalyze redox reactions (Santoro et al., 2016). Fifth, Cu(II) decreases proteasome activity in HeLa cells through ROS-mediated proteasome inhibition and disassembly of the 26S proteasome (Santoro et al., 2016). Finally, several copper complexes have strong anticancer activities by inhibiting the UPS *in vitro* and *in vivo* (Table 1). In this section, we will highlight several well-studied copper complexes in tumor therapy.

**TABLE 1:** Available copper complexes as UPS inhibitors.

Target	Agents	Tumor Models	Effects	Refs
20S proteasome	CuHQ	HL-60, Jurkat T, MCF10DCIS.com, and NK (YT) cells	Induces PARP1 cleavage, induces Ub accumulation, and increases BAX	Daniel et al. (2004), Zhai et al. (2010)
20S proteasome	CuCQ	MCF10DCIS.com and MDA-MB-231 cells	Induces PARP1 cleavage, induces Ub accumulation, and increases BAX	Daniel et al. (2005), Zhai et al. (2010)
20S proteasome	CuPDTC	MCF10DCIS.com, MDA-MB-231, and LNCaP cells	Induces PARP1 cleavage, induces Ub accumulation, and increases NFKBIA, CDKN1B, and BAX	Chen et al. (2005), Daniel et al. (2005), Yu et al. (2007), Milacic et al. (2008)
20S proteasome NPLOC4	CuET	MCF10DCIS.com, MDA-MB-231 cells, and U2OS cells; MDA-M-231 xenograft	Induces Ub accumulation, increases NFKBIA, CDKN1B, and BAX, and induces apoptotic and non-apoptotic cell death	Chen et al. (2006), Cvek et al. (2008), Skrott et al. (2017)
20S proteasome	Cu(I)ET	BXPC-3, PANC-1, and SW 1990 cells; SW 1990 cells xenograft	Induces Ub accumulation, increases CDKN1B, and deceases NFKB	Han et al. (2013)
20S proteasome	FPA-137	LNCaP and PC-3 cells	Induces PARP1 cleavage, and induces Ub accumulation	Adsule et al. (2006)
20S proteasome	Compound 1	Jurkat T and MDA-MB-231 cells	Induces PARP1 cleavage, induces Ub accumulation, and increases CDKN1B and BAX	Zhang (2008)
20S proteasome	GVC	Jurkat T, MCF10DCIS.com, and MDA-MB-231 cells	Induces PARP1 cleavage, induces Ub accumulation, and increases NFKBIA	Xiao et al. (2008)
20S proteasome	Cu(LI)Cl	C4-2B and Jurkat T cells	Induces PARP1 cleavage and induces Ub accumulation	Hindo et al. (2009)
20S proteasome	CuNC	Jurkat T and MDA-MB-231 cells	Induces PARP1 cleavage, induces Ub accumulation, and increases NFKBIA	Xiao et al. (2010)
20S proteasome	Complexes C1	MCF-7, MDA-MB-231, and PC-3 cells	Induces PARP1 cleavage, induces Ub accumulation, increases NFKBIA and BAX, and decreases XIAP	Zuo et al. (2013)
20S proteasome	Cu(OH-PIP) (Phe)Cl	CAL-51, MCF-7, and MDA-MB-231 cells	Induces PARP1 cleavage, induces Ub accumulation, increases BAX, and decreases pro-CASP3 and BCL2	Li et al. (2019b)
20S proteasome	Cu2(sal-D,L-glu)2 (isoquinoline)2	A549, HeLa, and U-118MG cells	Induces cytotoxicity	Konarikova et al. (2019)
UCHL5 and USP14	CuPT	HepG2, MCF-7, NCI-H929, SMMC-7721, and U266 cells; HepG2 xenograft	Induces PARP1 cleavage, induces Ub accumulation, increases CDKN1A, CDKN1B, BAX, and NFKBIA, and decreases pro-CASP8 and pro-CASP9	Liu et al. (2014a)
UCHL5 and USP14	CuHK	A549 and K562 cells	Induces PARP1 and CASP3 cleavage, induces Ub accumulation, increases ATF4, and induces paraptosis	Chen et al. (2017)

Abbreviations: ATF4, activating transcription factor 4; BAX, bcl2 associated X, apoptosis regulator; BCL2, bcl2 apoptosis regulator; CASP3, caspase 3; CASP8, caspase 8; CASP9, caspase 9; CDKN1A/p21, cyclin dependent kinase inhibitor 1A; CDKN1B/p27, cyclin dependent kinase inhibitor 1B; NFKBIA/IKBA, NFKB inhibitor alpha; NPLOC4, NPL4 homolog, ubiquitin recognition factor; PARP1/PARP, poly(ADP-ribose) polymerase 1; Ub, ubiquitin; UCHL5/UCH37, ubiquitin C-terminal hydrolase L5; USP14, ubiquitin-specific peptidase 14; XIAP, X-linked inhibitor of apoptosis.

### CuET

Disulfiram (DSF) is used to treat patients with alcohol dependence and has been repurposed for cancer treatment (McMahon et al., 2020). DSF is easily reduced to diethyldithiocarbamate (ET), which is a strong chelator of divalent metal ions. Formation of the DSF-copper (CuDSF) complex inhibits the chymotrypsin-like activity of the purified 20S or 26S proteasome in breast cancer cells (MDA-MB-231) (Chen et al., 2006). This selective inhibition of proteasome activity has been further confirmed in malignant MCF10DCIS.com breast cells, but not in normal MCF-10A breast cells (Chen et al., 2006). Addition of Cu(II) enhances anticancer activity of DSF by converting to bis(ET)-Cu(II) complex (CuET) (Cen et al., 2004). Interestingly, ZnET also has an effect of inhibiting proteasome activity similar to CuET (Cvek et al., 2008), although it is not clear whether they have the same structural basis. The activity of CuET on the cellular 26S proteasome is higher than that on purified 20S proteasome core particles (Cvek et al., 2008), indicating that CuET may mainly target 19S regulatory particles. In addition to inducing apoptosis, CuET also triggers non-apoptotic cell death, such as paraptosis in drug-resistant prostate cancer cells (Chen et al., 2018b). The anti-cancer activity of CuET or CuIET has been proven in multiple xenograft mouse models (including, but not limited to SW 1990, MDA-MB-231, and AMO-1 cancer cells) and has good tolerance (Han et al., 2013; Skrott et al., 2017; Peng et al., 2020). Moreover, some clinical trials of the combination of DSF and copper have been completed (NCT00742911 and NCT03034135) or are in progress (NCT04265274, NCT03714555, NCT03363659, and NCT02715609), providing a potential strategy for tumor therapy.

Inhibiting VCP segregase adaptor NPLOC4 is another appealing mechanism contributing to the anticancer activity of CuET (Skrott et al., 2017). Unlike NMS873 (a well-known inhibitor of ATPase activity of VCP), CuET has no effect on this ATPase activity (Skrott et al., 2017). Isothermal calorimetry analysis reveals that CuET can directly bind to NPLOC4, leading to NPLOC4 aggregation (Skrott et al., 2017). Consequently, ectopic overexpression of NPLOC4 reverses CuET-mediated cytotoxicity (Skrott et al., 2017), although the role of VCP in this process remains unknown. Similar to proteasome inhibitors (e.g., MG132 and bortezomib), CuET induces accumulation of ubiquitylated proteins and rapid deubiquitylation of histone H2A in MCF-7 cells (Skrott et al., 2017). In another case, CuET prevents degradation of NFKBIA and Ub(G76V)-GFP (a degradation substrate for proteasome) *in vitro* (Skrott et al., 2017). However, different from bortezomib or MG132, CuET induces a weak accumulation of hypoxia inducible factor 1 subunit alpha (HIF1A) (Skrott et al., 2017). These findings establish overlap and difference in substrate selection between CuET and classic proteasome inhibitors.

### CuHQ and CuCQ

The copper-containing compound NCI-109268 was identified as a proteasome inhibitor from a screening library of the National Cancer Institute (USA), which contains 1990 compounds (Daniel et al., 2004). Later, it was discovered that the bis(8-hydroxyquinoline)–Cu(II) complex (CuHQ) has a similar structure with NCI-109268, and its inhibitory effect on the CT-like activity is stronger than the T-like activity of proteasome (Daniel et al., 2004). CuHQ induces accumulation of ubiquitinated proteins and subsequent apoptotic death in Jurkat T cells (Daniel et al., 2004), suggesting potential anticancer activity. Moreover, increased cellular copper concentrations by a copper-enriched culture medium enhances sensitivity of prostate cancer PC-3 cells to 8-hydroxyquinoline (Daniel et al., 2004), indicating that exogenous copper can be used as a sensitizer for chemotherapeutics.

As a topical antifungal drug used clinically, clioquinol (5-chloro-7-iodo-8-hydroxyquinoline, CQ) is an analog of 8-hydroxyquinoline. CQ can form a stable complex with Cu(II), namely CuCQ, which exhibits potent cytotoxicity in human cancer cell lines (Khan et al., 2020; Wehbe et al., 2018). Mechanistically, CuCQ acts as an inhibitor of the CT-like activity of proteasome, leading to apoptotic death in MDA-MB-231 cells (Daniel et al., 2005). In particular, CQ-mediated cytoplasmic clearance of baculoviral IAP repeat containing 2 (BIRC2/CIAP1) and baculoviral IAP repeat containing 3 (BIRC3/CIAP2) contribute to CuCQ-induced apoptosis (Cater and Haupt, 2011). Similar to 8-hydroxyquinoline, copper-enriched cancer cells are also sensitive to CQ treatment (Daniel et al., 2005), further suggesting that formation of CuCQ can suppress tumor growth. Importantly, CQ inhibits the proteasome in leukemia and myeloma cells, but is not effective on normal cells (Mao et al., 2009), indicating that it has selective antitumor activity. Other analogs of CQ also inhibit the proteasomal activity and proliferation of cancer cells, such as human breast cancer (MCF10DCIS.com), ovarian cancer (A2780), and lung cancer (A549) cells (Zhai et al., 2010; Oliveri et al., 2017). However, the features recognized by these CQ analogs on the proteasome are largely unknown.

### CuPDTC

The mixture of pyrrolidine dithiocarbamate (PDTC) and copper inhibits proteasome function and induces cell death in human breast cancer (MDA-MB-231) and prostate cancer (LNCaP) cells (Chen et al., 2005; Daniel et al., 2005). The involvement of bis(PDTC)-Cu(II) (CuPDTC) in inhibiting the CT-like activity of proteasome is confirmed in MDA-MB-231 cells (Milacic et al., 2008). Moreover, in MDA-MB-231 cells, several tumor-related proteins (e.g., NFKBIA and CDKN1B) are identified as proteasome targets of CuPDTC (Milacic et al., 2008). It is unclear whether PDTC analog-driven proteasome inhibitors have similar protein substrate targets for tumor suppression (Yu et al., 2007; Wang et al., 2011). In addition, CuPDTC is also engaged in induction of protein cleavage during apoptosis. For example, in apoptosis induced by chemotherapeutics, caspase or calpain family proteins usually play a parallel role to cleave poly-ADP-ribose polymerase 1 (PARP1/PARP) to impair nuclear function (Pink et al., 2000). In MDA-MB-231 cells, CuPDTC induces calpain-dependent PARP1 cleavage and apoptosis, and this process is reversed by calpastatin (a calpain inhibitor) (Milacic et al., 2008). Whether CuPDTC-induced apoptosis depends on calpain (rather than caspase) needs to be further investigated in a variety of cancer cells. At least, in cisplatin-resistant neuroblastoma cells, CuPDTC can induce cell death and cell cycle arrest by increasing TP53 protein expression, which usually leads to caspase-dependent apoptosis (Zhang et al., 2008). Interestingly, similar to CuPDTC, ZnPDTC also triggers proteasome inhibition and PARP1 cleavage (Milacic et al., 2008), raising questions about whether and how CuPDTC and ZnPDTC bind to the 20S proteasome. Further research also needs to confirm the direct molecular link between CuPDTC-induced proteasome inhibition, apoptosis induction, and structural protein cleavage.

### CuPT

Pyrithione has a broad antimicrobial activity and excellent metal binding potential (Turley et al., 2000), whereas copper pyrithione (CuPT) not only has antifouling paint biocides (Almond and Trombetta, 2016), but also exerts potent anticancer activity (Liu et al., 2014a). CuPT inhibits cancer cell growth by targeting active sites of 19S DUBs (UCHL5 and USP14), leading to accumulation of both total and K48-linked ubiquitinated proteins (e.g., CDKN1A, CDKN1B, BAX, and NFKBIA) and GFPu (a surrogate proteasome substrate) (Liu et al., 2014b). Accordingly, CuPT inhibits DUB activity of the 26S proteasome in a cell-free system and can compete with UbVS (a potent inhibitor against UCHL5 and USP14) binding with UCHL5 and USP14 (Liu et al., 2014b). In contrast, although high doses may produce off-target effects, low doses of CuPT cannot prevent the CT-like activity of the 20S proteasome. The anticancer activity of CuPT is mediated by inducing apoptosis in various cancer cell lines (MCF-7, U266, and HepG2), primary monocytes from patients with acute myeloid leukemia, and xenograft mouse models (Liu et al., 2014b). These findings expand our understanding of extensive role of metal pyrithione complexes in inhibiting proteasomal DUBs (Zhao et al., 2016a; Zhao et al., 2016b; Zhao et al., 2017; Chen et al., 2018).

### CuHK

Hinokitiol is a tropolone-based phenolic component isolated from Cupressaceae heartwood and its anticancer effects and antimicrobial activity have been well-documented (Inoue et al., 2020; Wu et al., 2020). In addition, the hinokitiol copper complex (CuHK) has been confirmed as an inhibitor of the 19S DUB, but not the CT-like activity of the 20S proteasome (Chen et al., 2017). This function of CuHK on DUB inhibition causes accumulation of ubiquitinated proteins and GFPu in cancer cells (A549 and K562) and HEK293 cells, respectively (Chen et al., 2017). Consequently, CuHK triggers paraptosis-like cell death (Chen et al., 2017), a caspase-independent form of regulated cell death characterized by ER and/or mitochondria dilation (Fontana et al., 2020). In particular, activating transcription factor 4 (ATF4)-mediated ER stress, but not ROS generation, favors CuHK-induced paraptosis-like cell death in A549 and K562 cells (Chen et al., 2017). It remains to be defined whether CuHK-induced paraptosis is involved in activation of ERAD machinery in ER.

## Conclusion and Perspectives

The essential role of the UPS in controlling cancer biology has aroused great interest in the development of proteasome inhibitors as anticancer drugs. In recent years, tremendous progress has been made in understanding the potential of copper complexes as anticancer drugs by inhibiting multiple components and regulators of the UPS, such as 20S proteasome, 19S DUBs, and NPLOC4, although it is expected that E1, E2, and E3 might also be their targets. A main challenge in the future is to reveal precise mechanisms and specific substrate aspects of UPS inhibition by copper complexes. It is also important to profile activity and side effects of copper complexes versus other metal-containing drugs on inhibiting protein degradation and inducing different cell death modalities, toward developing them as clinically used anticancer agents. In addition, it will be interesting to distinguish between UPS-dependent and UPS-independent anticancer activities of copper complexes. A systematic and rigorous research on drug structure and function may help in addressing these issues. However, severe side effects and poor target specificity remain major obstacles for the development of copper complexes as anticancer drugs. Various nanoparticles may help provide better targeting and drug delivery of metal complexes for multiple tumor models (Sharma et al., 2018). It is also important to improve target selectivity by modifying the ligand of copper complexes and investigating the structure-activity relationship.
